# Retrospective mortality among refugees from the Central African Republic arriving in Chad, 2014

**DOI:** 10.1186/s13031-017-0110-4

**Published:** 2017-05-15

**Authors:** Matthew E. Coldiron, Thomas Roederer, Augusto E. Llosa, Malika Bouhenia, Sassou Madi, Laurent Sury, Michaël Neuman, Klaudia Porten

**Affiliations:** 1 Epicentre, 8 rue Saint-Sabin, Paris, France; 2 Médecins Sans Frontières, Ndjaména, Chad; 3 Médecins Sans Frontières, 8 rue Saint-Sabin, Paris, France; 4 CRASH, 8 rue Saint-Sabin, Paris, France

**Keywords:** Refugees, Mortality, Central African Republic, Chad, Violence, Exposure to violence, War crimes

## Abstract

**Background:**

The Central African Republic has known long periods of instability. In 2014, following the fall of an interim government installed by the Séléka coalition, a series of violent reprisals occurred. These events were largely directed at the country’s Muslim minority and led to a massive displacement of the population. In 2014, we sought to document the retrospective mortality among refugees arriving from the CAR into Chad by conducting a series of surveys.

**Methods:**

The Sido camp was surveyed exhaustively in March-April 2014 and a systematic sampling strategy was used in the Goré camp in October 2014. The survey recall period began November 1, 2013, just before the major anti-Balaka offensive. Heads of households were asked to describe their household composition at the beginning o﻿f and throughout the recall period. For household members reported as dying, further information about the date and circumstances of death was obtained.

**Results:**

In Sido, 3449 households containing 25 353 individuals were interviewed. A total of 2599 deaths were reported, corresponding to a crude mortality rate of 6.0/10000 persons/day, and 8% of the population present at the beginning of the recall period died. Most (82.4%) deaths occurred among males, most deaths occurred in December 2013 and January 2014, and 92% were due to violence in the CAR.

In Goré, 1383 households containing 8614 individuals were interviewed. A total of 1203 deaths were reported, corresponding to a crude mortality rate of 3.7/10000 persons/day [95%CI 3.5–3.9], and 12% of the population present at the beginning of the recall period died. Most (77.1%) deaths occurred among males. As in Sido, most deaths occurred in December 2013 and January 2014, and 86% of all deaths were due to violence in the CAR.

**Conclusions:**

The results of these two surveys describe a part of the toll of the violent events of December 2013 and January 2014 in the Central African Republic.

## Background

In 2003, General Francois Bozizé became the President of the Central African Republic (CAR) in a coup d’etat. Repeated bouts of armed conflict began around the country in 2004 and despite peace agreements with some groups in 2007 and 2011, more fighting broke out in December 2012, after the Séléka coalition was formed in opposition to President Bozizé. What began as a political movement also took on both ethnic and religious aspects.

The majority-Muslim Séléka eventually took control of the country for several months, taking Bangui in March 2013 [1]. The new regime was responsible for many violent acts, and some considered that a genocide was imminent [2]. They were eventually driven from power by a coalition of opposition militias known as the anti-Balaka. The anti-Balaka groups made major advances at the end of 2013 and beginning of 2014, and violence targeting the Muslim minority led to massive population displacement [3]. Estimates from the summer of 2013 reported over 200,000 internally displaced persons and similar numbers of refugees forced to flee CAR to neighboring countries [4]. In response to these events, two international peacekeeping forces were deployed in the CAR in December 2013: a United Nations force called the International Support Mission to the Central African Republic (MISCA) and a French force called Operation Sangaris.

Documenting the number of deaths resulting from these violent incidents is highly problematic, particularly as these events could be considered crimes against humanity, if indeed specific civilians were targeted for death and depravation due to their membership in an ethnic or religious group [5]. In order to provide an estimate of the number of deaths among CAR refugees who fled to Chad during the most acute phase of the crisis in 2013, we conducted retrospective mortality surveys in camps located in Goré and Sido, Chad. The aim of these surveys was to estimate the crude mortality, to describe where deaths occurred, and to describe how many were due to violence.

## Methods

From 26 March – 8 April 2014, an exhaustive household survey was carried out among displaced persons living in, Sido, Moyen-Chari, Chad. At the time of the survey, some displaced persons were registered and living in a semi-organized camp, others were living in informal settlements around Sido town in an area covering approximately 12 km^2^. The camp was divided into sectors, each of which was assigned to a member of survey staff. As some displaced persons were living in tents or rented rooms among the host population in Sido, each household of the town was also visited. After a household had been visited by the member of survey staff, a small mark was made on an outer wall or piece of plastic sheeting to indicate that the residents had already been included in the survey.

From 23 – 30 October 2014, a household survey was carried out among displaced persons living in two camps around Goré, Logone Orientale, Chad. At the time of the survey, 2912 households were officially registered in the Danamadja camp and 1675 in the Kobiteïe camp. Zones of the camp were clearly delimited, and systematic random sampling was used. The target sample size (1336 households) was set in order to be able to estimate a mortality rate of 9/10000 persons/day over the two-month period covering December 2013 and January 2014 with a precision of 1/10000/day around the point estimate.

The recall for both surveys began 1 November 2013, celebrated locally as the day of the dead (All Saints’ Day), and occurring just prior to the main Séléka offensive towards Bangui. The median recall period for the surveys was therefore 153 days in Sido and 360 days in Goré.

In both surveys, in each household visited, the head of household was asked a series of questions regarding the composition of his or her household during the entire recall period. A household was defined as a group of people who shared meals and slept in the same place at any point during the recall period, under the authority of a single head of household. Kinship ties were not taken into account, and those present in the household for a limited period of time during the recall period were included for that period of time. Those who left during the recall period were classified as having left voluntarily, as having left involuntarily but with recent information that the person was alive, as having disappeared (no information about the person’s vital status), or as having died. For those reported as dead, information was collected regarding the date, place of death (at home, in transit, or in camp) and cause of death (violence, illness or accident). Details were collected regarding the circumstances of violent deaths. Precise dates for each event (births, deaths, separations, and arrivals) were collected and used in the calculations of person-time.

Heads of household were also asked about their usual home in the CAR, the date of their departure from their usual home and arrival to intermediate destinations and the camps in Chad.

Heads of household above the age of majority provided their consent prior to participation in the survey. A text was read to potential respondents stating the aims of the survey, underscoring the voluntary nature of the survey, and clearly stating that the choice to participate or not would have no impact on access to health care or food distributions. Households for which the head of household was a minor were not included in the surveys. The survey protocol was reviewed and approved by the National Bioethical Committee of Chad and by the Ministry of Health of Chad.

## Results

**Fig. 1:**
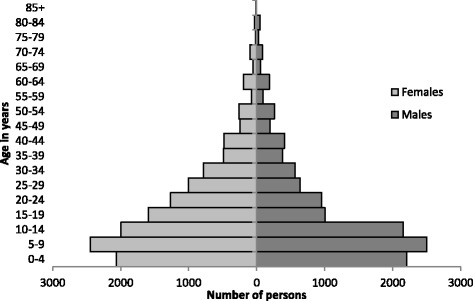
Population pyramid, refugees present at Sido, Tchad, March-April 2014

**Fig. 2:**
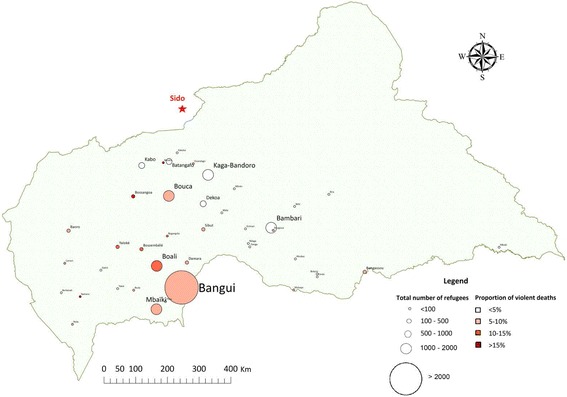
Origin of refugee households present in Sido, Chad, October 2014

In Sido, 3449 households were interviewed, containing 25 353 individuals (average household size 7.4 persons), including 4266 children under 5 years old (16.8% of the total population). There were no refusals to participate in the survey. Females represented 52% of the population present in the camp. Figure 1 shows the population pyramid of persons present in the camp, showing an underrepresentation of males aged 15–39 years compared to females of the same age group. A total of 57% of refugees present in Sido lived in Bangui before they left the CAR (Fig. 2). The median length of time between leaving home and arriving in Sido was 4 days [IQR 3–7, min 1, max 137], and 85.3% arrived in January or February 2014. At the beginning of the recall period, a total of 32 346 persons were present in the households, and Table 1 presents the evolution of the population over the recall period.

**Table 1:** Evolution of population of refugee households in Sido and Goré, Chad, November 2013-April 2014

	Sido	Camps near Goré
Persons present in household on 1 November 2013	32 346	10 090
Voluntary separation	715	58
Involuntary separation but known to be alive	3751	400
Involuntary separation and vital status unknown	350	336
Known deaths	2599	1203
* Total Departures*	7415	1997
Newborns	255	254
New household members joined	167	267
* Total arrivals*	422	521
Total present at time of survey^a^	25 353	8614

^a^Survey date in Sido from 26 March-8 April 2014, survey date in Goré 23–30 October 2014

A total of 2599 deaths were reported during the recall period, corresponding to a crude mortality rate (CMR) of 6.0 deaths per 10000 persons per day. Overall, 8% of the population present on November 1, 2013, died during the recall period, including 12.1% of men. A total of 120 deaths occurred in children aged under 5 years, corresponding to an age-specific mortality rate among children under 5 (U5MR) of 1.8 deaths / 10000 persons / day. 2143 deaths (82.4%) occurred among males, leading to a sex-specific mortality rate of 9.6 deaths / 10000 persons / day over the recall period. When considering households, 33.0% of respondent households had at least one member die, and 27.6% of households had at least two members die during the recall period.

**Fig. 3:**
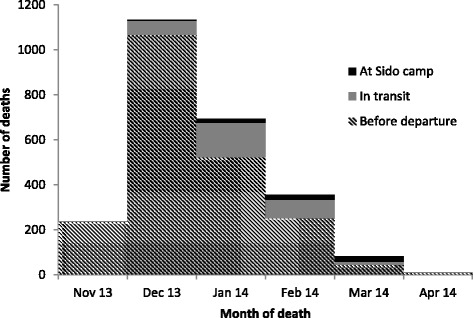
Deaths per month, refugee households present in Sido, Chad, March-April 2014

Figure 3 shows the number of deaths per month, with 1882 deaths (74.2%) occurring in December 2013 and January 2014. Table 2 describes the circumstances of death. A total of 2208 deaths (85.0%) occurred before leaving CAR (CMR 9.1/10 000 persons/day), but when considering the time periods, the highest mortality rates occurred during transit (13.5/10 000 persons/day). 91% of all deaths were violent, including 1998 of 2143 deaths among males and 367 of 446 deaths among females. Among violent deaths, 51% were reported as being due to gunshot and 47% due to small weapons. The remaining deaths were reported as being due to beatings and other causes. Among deaths occurring in the CAR prior to departure from home, 2110 (96%) were reported as being due to violence, as were 252 (78%) of deaths occurring during transit. After arrival in Sido, only 3 (4%) of reported deaths were due to violence.

**Table 2:** Deaths and mortality rates by sex, Sido, Chad, 2014

	Males		Females		Overall	
Deaths	Number	Mortality Rate (/10000/day)	Number	Mortality Rate (/10000/day)	Number	Mortality Rate (/10000/day)
Before departure	1863	14.3	345	3.1	2208	9.1
In transit	241	20.2	71	6.7	322	13.5
At Sido camp	39	0.5	30	0.3	69	0.4
Total	2143	9.6	446	2.1	2599	5.9
Cause of death	Number	% of total	Number	% of total	Number	% of total
Violence	1998	93.2	367	80.5	2365	91.0
Illness	82	3.8	52	11.4	134	5.2
Accident	35	1.6	32	7.0	67	2.6
Unspecified	28	1.3	5	1.1	33	1.3

**Fig. 4:**
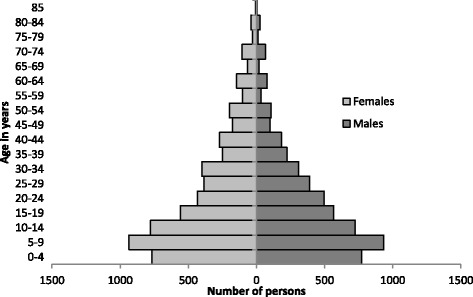
Population pyramid, refugees present at Goré, Tchad, October 2014

**Fig. 5:**
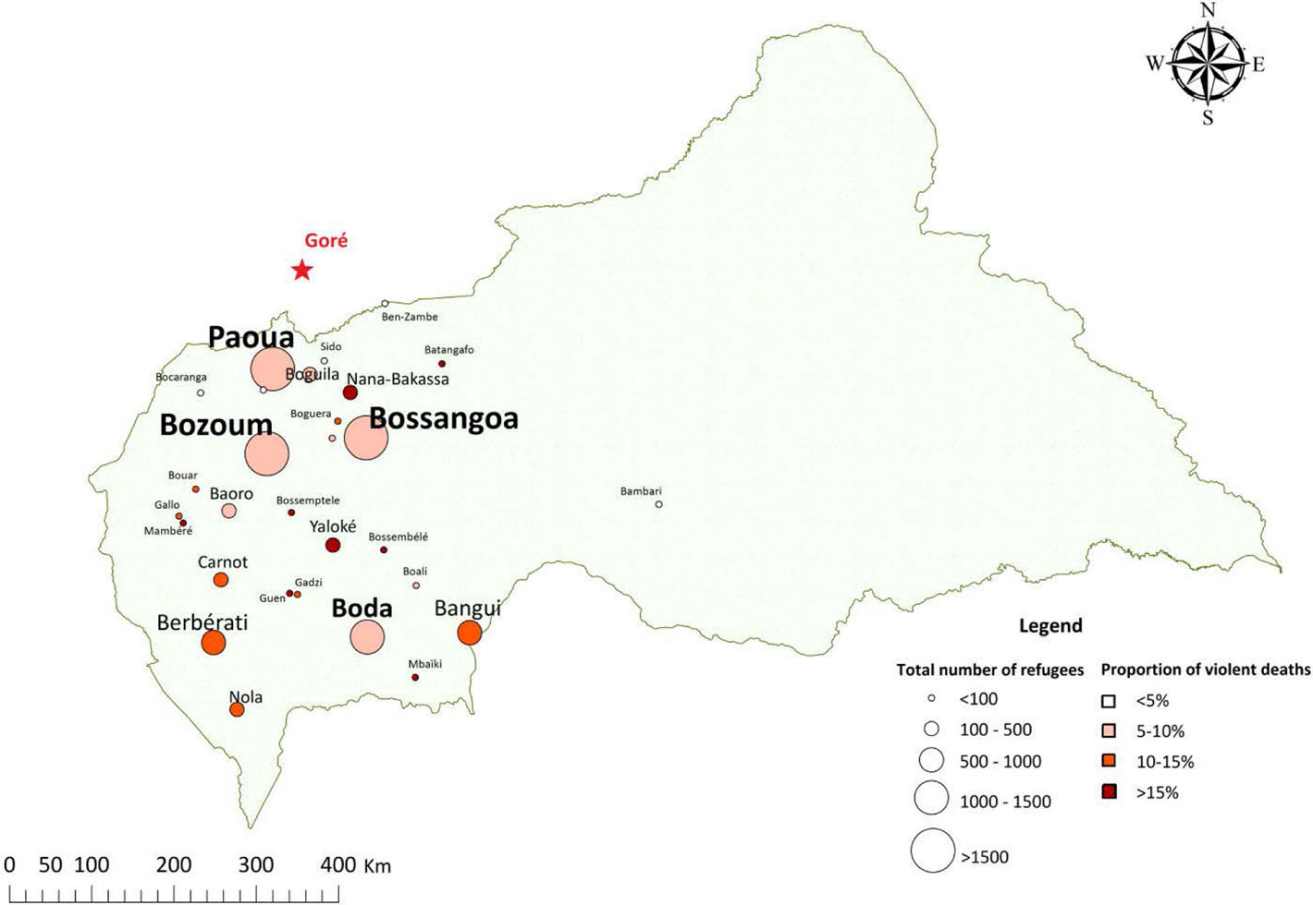
Origin of refugee households present in Goré, Chad, October 2014

In Goré, 1383 households were interviewed, containing 8614 individuals (average household size 6.2 persons), including 1399 children under 5 years old (16.2% of the total population). Females represented 50% of the population present in the camp. Heads of household were asked to report their religion; 98% were Muslim. Figure 4 shows the population pyramid of persons present in the camp, showing an underrepresentation of males aged 20–39 years compared to females of the same age group. The population present in Goré lived in a variety of locations around the CAR, but more lived in the western part of the country, and fewer came from Bangui compared with the residents of Sido camp (Fig. 5). The median length of time between leaving home and arriving in Goré was 131 days [IQR 108–184, min 32, max 1246], and 59.9% arrived in April or May 2014, with a second wave arriving in August 2014 (36.0% of the population at the time of the survey). At the beginning of the recall period, a total of 10 090 persons were present in the households, and Table 1 presents the evolution of the population over the recall period.

A total of 1203 deaths were reported during the recall period, corresponding to a CMR of 3.7 deaths / 10000 persons / day (IC95% [3.5–3.9]). Overall, 12% of the population present on November 1, 2013 died during the recall period, including 16.4% of men. A total of 95 deaths occurred in children aged under 5 years, corresponding to an U5MR of 1.8 deaths / 10000 persons / day (IC95% [1.5–2.2]). 926 deaths (77.1%) occurred among males, leading to a sex-specific mortality rate of 5.8 deaths / 10000 persons / day over the recall period. When considering households, 49.1% of respondent households had at least one member die, and 21.0% of households had at least two members die during the recall period.

**Fig. 6:**
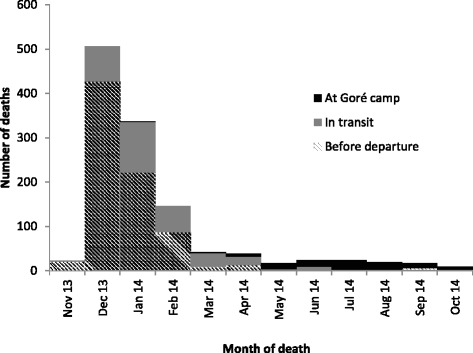
Deaths per month, refugee households present in Goré, Chad, October 2014

Figure 6 shows the number of deaths per month, with 1718 deaths (83.4%) occurring between December 2013 and February 2014. Table 3 describes the circumstances of death. A total of 1105 deaths (91.9%) occurred before leaving CAR or during transit. A total of 86% of all deaths were violent, including 828 of 926 deaths among males and 211 of 277 deaths among females. Among deaths occurring in the CAR prior to departure from home, 746 (95%) were reported as being due to violence, as were 290 (91%) of deaths occurring during transit. After arrival in the Goré camps, only 3% of reported deaths were due to violence.

**Table 3:** Deaths and mortality rates by sex, Goré, Chad, 2014

	Males		Females		Overall	
Deaths	Number	Mortality Rate (/10000/day) [95% CI]	Number	Mortality Rate (/10000/day) [95% CI]	Number	Mortality Rate (/10000/day) [95% CI]
Before departure	628	18.7 [17.3–20.1]	158	5.0 [4.2–5.8]	786	12.1 [11.3–12.9]
In transit	244	1.1 [0.8–1.4]	75	0.3 [0.1–0.4]	319	0.7 [0.5–0.8]
At Goré camp	54	0.5 [0.3–0.7]	44	0.4 [0.2–0.5]	98	0.4 [0.3–0.6]
Total	926	12.9 [11.7–14.2]	277	4.7 [4.0–5.5]	1203	8.8 [8.1–9.6]
Cause of death	Number	% of total	Number	% of total	Number	% of total
Violence	828	89.4	211	76.1	1039	86.4
Others	98	10.6	66	23.9	164	12.6

## Discussion

In these two surveys, we have described a surge of deaths among refugee households fleeing the CAR during December 2013 and January 2014. In raw figures, we have reported 3802 deaths among households present in two refugee camps. The vast majority of deaths in these households were reported as being due to violence, and most of them occurred before the household fled their home, or in transit to their refugee camp. Young adult males were most affected.

At the time of these events, there was much discussion over the correct terminology to use to describe the events (civil war, targeted killings, religious-based violence, and genocide were all used). The results of two retrospective mortality surveys cannot definitively characterize these events, but it is clear that the violence was widespread and differentially affected young Muslim men.

The populations of the two camps surveyed were quite different, with those in Sido being mostly from Bangui, and arriving quickly after the main peak of violence, while in Goré, the population came from around the CAR, and had spent a much longer period of time in transit to reach the refugee camps. The patterns of mortality were dissimilar among refugees in both camps: for those in Sido, the highest rates were seen during transit, followed by the time before departure from home. In contrast, for those in Goré, the highest rates were seen before leaving home. The low mortality rates during transit seen among refugees living in Goré are likely explained by the increased length of time in transit. In both cases, deaths in transit were largely reported as being caused by violence. This result correlates with reports at the time that the convoys transporting refugee families were often targeted en route.

In presenting these results, we have described mortality both in terms of mortality rates, a standard measure during humanitarian emergencies, as well as in terms of absolute numbers of deaths. But we cannot further extrapolate these results: the surveys were not representative of all households who fled the CAR, only those in Sido and Goré. Those arriving in other camps in Cameroon, the Republic of Congo and the Democratic Republic of Congo may have come from different places at different times and had different experiences. Likewise, these surveys did not take into account the households remaining in the CAR that also suffered violent events, including those of the Christian majority.

But the proportions we report provide a glimpse of the pervasiveness of the violence, with a third of all households in Sido and nearly a half of all households surveyed in Goré reporting at least one death. We would argue that in similar situations, absolute numbers and percentages are actually more powerful descriptors of the events than the standard calculated mortality rates, which are compared to standard thresholds. In either case, the crude mortality rates largely surpass the commonly-used threshold of 1 death/10 000 persons/day used to define a humanitarian emergency.

The fact that the survey in Sido was an exhaustive survey occurring soon after the arrival of most refugees is a major strength of our analyses, as was the fact that it included refugees living inside the host community. And while we believe that the information was truly exhaustive (no refusals to participate and a system of marking already-surveyed shelters), given that the survey happened during the acute phase of an emergency situation in a non-organized refugee camp, it is possible that some persons living in Sido at the time of the survey were not included. The length of the recall period in the Goré survey was nearly one year, which does introduce some possibility for recall bias, but we are reassured by the similar trends of results with refugees living in Sido.

The events of December 2013 and January 2014 in the CAR played out at the same time as the arrival of international armed forces and therefore drew a moderate amount of international attention, but the reaction was not swift enough to stop the violence that we have partially described in these surveys. We hope that by adding quantitative figures, in terms of mortality rates and numbers of deaths among refugee households, we can complement already-existing qualitative descriptions of this humanitarian emergency.

## Conclusions

We describe a total of 3802 deaths occurring in households that fled the CAR during the violent events of December 2013 and January 2014. The majority of these deaths were due to violence, and young men were particularly affected. These quantitative surveys complement qualitative research and the information made available in the popular press at the time of the events.

## Abbreviations

CAR: Central African republic
CMR: Crude mortality rate
IQR: Interquartile range
U5MR: Under-5 mortality rate
